# The Anatomic Relationship of the Tibial Nerve to the Common Peroneal Nerve in the Popliteal Fossa: Implications for Selective Tibial Nerve Block in Total Knee Arthroplasty

**DOI:** 10.1155/2017/7250181

**Published:** 2017-02-02

**Authors:** Eric R. Silverman, Amaresh Vydyanathan, Karina Gritsenko, Naum Shaparin, Nair Singh, Sherry A. Downie, Boleslav Kosharskyy

**Affiliations:** ^1^Department of Anesthesiology, Albany Medical Center, Albany, NY, USA; ^2^Department of Anesthesiology, Montefiore Medical Center, Bronx, NY, USA; ^3^Albert Einstein Medical College, Bronx, NY, USA

## Abstract

*Background.* A recently described selective tibial nerve block at the popliteal crease presents a viable alternative to sciatic nerve block for patients undergoing total knee arthroplasty. In this two-part investigation, we describe the effects of a tibial nerve block at the popliteal crease.* Methods.* In embalmed cadavers, after the ultrasound-guided dye injection the dissection revealed proximal spread of dye within the paraneural sheath. Consequentially, in the clinical study twenty patients scheduled for total knee arthroplasty received the ultrasound-guided selective tibial nerve block at the popliteal crease, which also resulted in proximal spread of local anesthetic. A sensorimotor exam was performed to monitor the effect on the peroneal nerve.* Results.* In the cadaver study, dye was observed to spread proximal in the paraneural sheath to reach the sciatic nerve. In the clinical observational study, local anesthetic was observed to spread a mean of 4.7 + 1.9 (SD) cm proximal to popliteal crease. A negative correlation was found between the excess spread of local anesthetic and bifurcation distance.* Conclusions.* There is significant proximal spread of local anesthetic following tibial nerve block at the popliteal crease with possibility of the undesirable motor blocks of the peroneal nerve.

## 1. Introduction

Arthroplasty of the knee is associated with moderate to severe postoperative pain [1]. Peripheral nerve blocks for total knee arthroplasty (TKA) are effective for postoperative pain management and may hasten the recovery process [2]. There are a number of regional techniques that have been used for TKA. A combination of the femoral nerve (FN) block and sciatic nerve (SN) block is an accepted technique for the postoperative pain management after TKA. Although the evidence for adding the SN block to FN block is controversial, it is commonly performed for TKA in many institutions [3–7].

Addition of the SN block may provide superior analgesia but can produce foot-drop or weakness of the tibialis anterior muscle due to blockade of the common peroneal nerve (CPN), which may mask surgically induced CPN injury [8]. The reported incidence of CPN injury after TKA ranges from 0.3% to 2.2% [9–15]. Potential risk factors for CPN injury include severe valgus deformity, preexisting neuropathy, rheumatoid arthritis, prolonged tourniquet time, and constrictive dressings. However, no single risk factor has been consistently shown to be associated with CPN [16].

Anatomical variation in the location where the SN bifurcates may predispose patients to complete blockade following selective TN block. In this combined anatomic investigation and prospective observational study, we aim to observe and describe the effects of this block. We hypothesize that proximal spread of local anesthetic towards the CPN occurs following selective TN block and accounts for partial CPN block. Secondly, we postulate that the more distal the bifurcation occurs, the more likely there will be spread from the popliteal injection site to the common sciatic nerve, resulting in unintended CPN blockade.

## 2. Methods

### 2.1. Anatomical Study

Approval was obtained from the local institutional review board at Albert Einstein College of Medicine. Four adult embalmed cadavers were used in this study. Each cadaver was placed in the prone position. Using a 13 to 6 MHz linear transducer (HFL38, SonositeTM, Bothell, WA), the tibial nerve (TN) was identified at the popliteal crease. Using a short-axis in-plane approach, a 50 mm, 21-gauge, stimulating needle (Arrow International, Teleflex Medical, Research Triangle Park, NC, USA) was advanced in a lateral to anteromedial direction toward the TN and 10 mL of blue dye (1% methylene blue diluted 1 : 10 with sterile water) was injected to encircle the target nerve. Subparaneural spread of local anesthetic was achieved using a technique similar to that described by Andersen et al. (2012) but at the level of the popliteal crease [17]. Immediately after injection, vertical skin incisions were performed over the popliteal fossa. The biceps femoris, semitendinosus, and semimembranosus muscles were carefully dissected to reveal the sciatic nerve and its branches. The sciatic nerve bifurcation was identified and the distance from the popliteal crease was recorded. If needed, the skin incision and muscle dissection were extended to identify the most proximal spread of dye within the paraneural sheath.

### 2.2. Case Series

Next, an observational prospective study on patients undergoing TKA was conducted. This investigation was also approved by the local institutional review board (Albert Einstein College of Medicine). The requirement for written informed consent was waived by the institutional review board and verbal consent was acceptable for data collection. All patients scheduled for primary total knee arthroplasty were candidates for this observational study. Exclusion criteria included age younger than 18 years, inability to communicate with the investigators or hospital staff, preexisting valgus deformity, flexion contracture or history of allergic reaction to amide local anesthetics, or patient refusal.

Preoperatively, intravenous access was secured, standard noninvasive monitors were applied, and supplemental oxygen was administered via nasal cannula. Patients were placed in the supine position and intravenous fentanyl and midazolam were titrated for patient comfort. Immediately prior to performing the block, thigh length and circumference were measured. Thigh length was recorded as the distance from inguinal crease to patella tendon insertion. A high-frequency linear array ultrasound transducer (SonoSite M-Turbo, Bothell, WA) was utilized for the identification of relevant landmarks and a continuous adductor canal block was performed using a mid-thigh approach described by Jæger et al. [18], whereas the needle positioning was accomplished under ultrasound guidance using a subsartorial approach with the visible local anesthetic spread around the femoral artery.

After the adductor canal catheter was placed, the lower extremity was flexed at the knee joint. By tracing the TN proximally, the convergence of the CPN and TN was identified and the distance from the convergence to popliteal crease was measured and recorded. A selective TN block was performed at the popliteal crease using a technique similar to that described by Andersen et al. (2012) but at the level of the popliteal crease [17]. This was the same approach as used in the anatomic study. Ten milliliters of 0.5% ropivacaine was injected. Immediately following injection, the bifurcation of the SN was reidentified and traced proximally to identify the extension of local anesthetic around the target nerves. Since local anesthetic was intended to encircle the TN and not the SN, local anesthetic extension proximal to the bifurcation was referred to as excess spread. The distance between the bifurcation and proximal extension of local anesthetic was recorded. Values were positive if spread extended proximal to the bifurcation and negative if spread extended but did not reach the bifurcation. For quality control, images were obtained and reviewed by a second expert in regional anesthesia.

Twenty minutes following TN block, tibial and peroneal sensorimotor functions were recorded. Tibial motor function (plantar flexion of the foot) and peroneal motor function (dorsiflexion of the foot) were tested with a 3-point scale (0 = normal, 1 = weak, and 2 = absent). Cold sensation was tested using an alcohol swab. Tibial sensation (plantar surface of the foot) and peroneal sensation (dorsal surface of the foot) were tested. Sensation was tested using a 3-point scale (0 = normal, 1 = absent cold perception, but touch sensation intact, and 2 = absence of touch sensation), as described in the study by Sinha et al. [8].

### 2.3. Statistical Analysis

An a priori sample size calculation is absent from the present observational study because the aim was to collect data for a larger investigation. A convenience sampling method was used to recruit patients into the study. The measurements obtained were plotted and analyzed using Prism 6.04 (GraphPad Software, Inc., San Diego, CA, USA). Spearman's rank correlation coefficient was applied for continuous data with *P* < 0.05 considered statistically significant. Results of the sensorimotor exams were reported and described and statistical analysis was not performed.

## 3. Results

### 3.1. Anatomical Study

**Figure 1:**
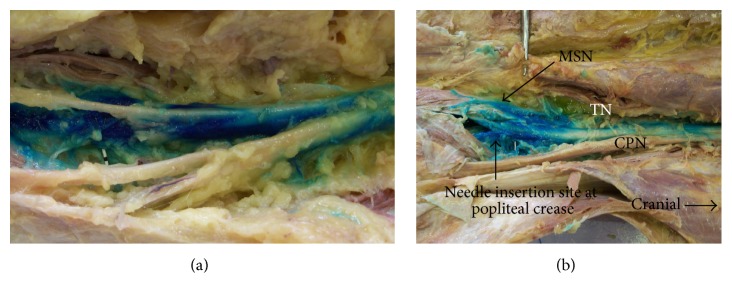
Blue dye injected around the tibial nerve at the popliteal crease. (a) Spread of blue dye is observed with significant extension proximal to the bifurcation point of the sciatic nerve. Complete sparing of the common peroneal nerve is observed at the level of the popliteal crease, (b) MSN = medial sural nerve; TN = tibial nerve; CPN = common peroneal nerve; SN = sciatic nerve.

Six injections and dissections, in 4 embalmed cadavers, were performed. Two limbs from two separate cadavers were not suitable for the procedure due to incidental finding of femoral fracture in one cadaver and a large venous thrombosis in the second. There were 3 males and 1 female with a mean age of 80 years (range 58–93 years). In all cases, the SN and its branches were easily visualized with ultrasound and paraneural injection was achieved. Following dissection, blue dye was observed to be contained within the paraneural sheath of the tibial nerve with only minimal extravasation to adjacent structures, Figure 1(a). There was no blue dye observed around the CPN at the level of injection; however, dye was observed extending proximal to the SN bifurcation, Figure 1(b). The mean distance from SN bifurcation to the popliteal crease was 6.6 cm (range 4.5–9 cm). Dye was observed to spread 14.5 cm (range 8–21 cm) proximal to the popliteal crease. Excess spread beyond the bifurcation occurred in all specimens and was observed to extend a mean of 7.9 cm (range 1.6–14 cm) proximal to the bifurcation.

### 3.2. Case Series

**Figure 2:**
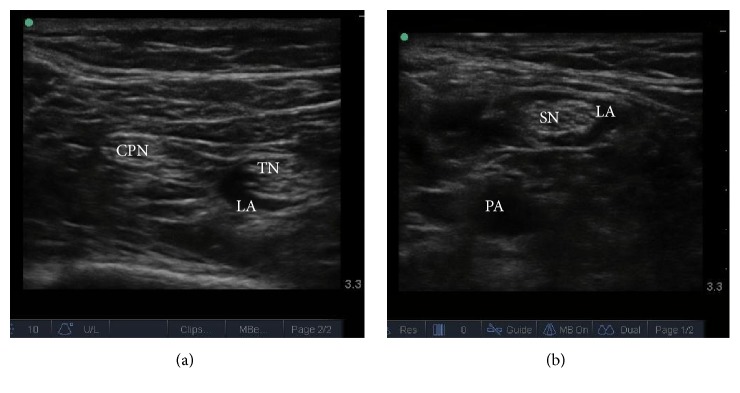
Ultrasound image of the common peroneal nerve and tibial nerve at the popliteal crease after selective tibial nerve block. Local anesthetic was observed around the tibial nerve but not the common peroneal nerve. (a) Local anesthetic could be traced proximally toward the bifurcation which was observed to encircle the sciatic nerve proximal to the bifurcation, (b) CPN = common peroneal nerve; TN = tibial nerve; LA = local anesthetic; SN = sciatic nerve; PA = popliteal artery.

**Figure 3:**
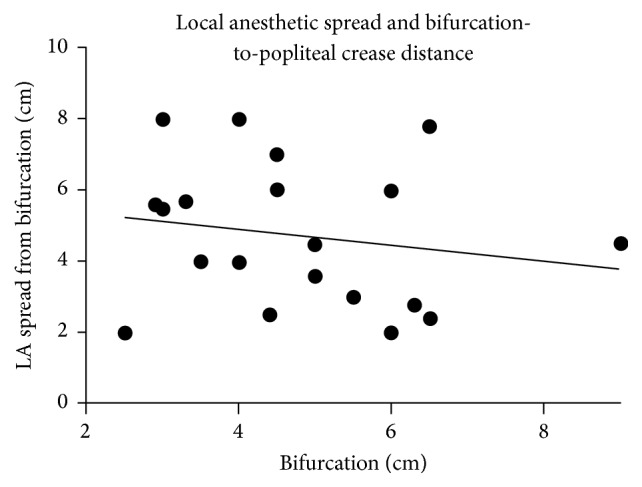
Scatter plot showing the relationship between bifurcation length and excess spread of local anesthetic. Excess spread was measured as the distance between the bifurcation point and maximal spread of local anesthetic proximal to the bifurcation. A negative correlation was found between the excess spread of local anesthetic and bifurcation length, *r* = −0.5605, *p* = 0.01.

Twenty patients were included in the analysis. Demographic and biometrical data of the study subjects are shown in Table 1. In every subject, the SN and its branches, CPN and TN, were visualized and could be traced back to the proximal bifurcation using the ultrasound probe. The bifurcation was found to be 4.8 ± 1.6 (SD) cm proximal to the popliteal crease. After selective TN block was performed, the local anesthetic could be traced proximally toward the bifurcation without difficulty in all patients. At the level of the popliteal crease, local anesthetic encircled the TN, but not the CPN, Figure 2. Mean spread was 4.7 ± 1.9 (SD) cm proximal to popliteal crease and extended proximal to the bifurcation in 9 cases. Using a two-tailed Spearman's correlation rank coefficient, a negative correlation was found between the excess spread of local anesthetic and bifurcation length, *r* = −0.5605, *p* = 0.01, Figure 3. No significant correlation was observed between bifurcation length and thigh length (−0.4383, *p* = 0.0532).

### 3.3. Sensorineural Examination

Sensory block in the distribution of the TN was observed in 95% of the patients. Of these, 14 patients had partial sensory block (sensory score of 1) while 5 had complete sensory block (sensory score of 2). In the distribution of the CPN, sensory block was observed in 75% patients. Of these 12 had a partial sensory block and 3 had a complete sensory block.

Motor block in the distribution of the TN was observed in 70% of patients. Of these, 12 had a partial motor block (motor score of 1) and 2 had a complete motor block (motor score of 2). Motor block of the CPN was observed in 15% (3 patients); however, complete motor block was not observed.

## 4. Discussion

Proximal spread of local anesthetic occurs following selective TN block at the popliteal crease. This was observed both in embalmed cadavers and in human subjects. In the clinical case series, a statistically significant negative correlation was observed between bifurcation length and spread above the bifurcation (*p* = 0.01), such that a shorter bifurcation length correlated with a greater degree of local anesthetic extension proximal to the bifurcation. Interestingly, in the anatomic study, blue dye was observed to extend within the paraneural sheath to a much greater degree than that seen under ultrasound in the case series (mean 14.5 cm compared with 4.7 cm, resp.). This was likely due to differences in the injectate dynamics between embalmed cadavers and living tissue. It is difficult to draw any correlations between these findings which is a limitation of this investigation.

A recent randomized-controlled trial by Sinha et al. (2012) demonstrated the feasibility of performing an ultrasound-guided selective tibial nerve (TN) block to avoid complete peroneal motor block. In the study involving 80 patients undergoing TKA, patients were randomized to receive a traditional SN block or selective tibial nerve block at the popliteal crease [8]. The authors reported similar pain scores and 24-hour opioid consumption across study arms. None of the patients who received the selective TN block developed complete motor block; however, nearly a quarter of the patients developed partial peroneal motor block. The authors postulated that proximal spread of local anesthetic from the site of blockade could have been responsible for the sensory block involving the CPN, but this was not investigated. Performing a selective TN block may decrease the likelihood of masking a surgically induced foot-drop compared with SN block; however, partial blockade of the CPN may be undesirable when a thorough postoperative neurologic exam is needed.

We confirm some of the findings reported by Sinha et al. (2012) in which a similar technique and local anesthetic dose were used. We observed similar rates of block success, 75% compared with 95% by Sinha et al. Three patients had reduced motor strength in the distribution of the CPN and none had complete motor block. Reduced motor strength of the CPN was consistent with the previously published report (15% compared with 22.5%, resp.). No patients developed complete CPN motor block in either study.

Our findings on bifurcation distance are supported by the literature [19]. In an anatomic investigation using MRI, the TN and CPN were found to divide at 5.8 cm with a range from 3.6 cm to 12.4 cm above the popliteal crease [19]. In our study, median distance was 4.8 cm with a range from 2.5 cm to 9 cm. A shorter bifurcation length correlated with statistically significant spread above the bifurcation, *r* = −0.5605 (*p* < 0.01). While the present study was not powered to detect a correlation between bifurcation length and degree of CPN block, we observed that local anesthetic almost always extended above the bifurcation when it was less than 5 cm from the popliteal crease.

Since the report published by Sinha et al. on the selective tibial nerve block we have adopted the technique to our practice and have observed excellent satisfaction from both patients and our surgeons. Despite this, we continue to observe a subset of patients that develop partial CPN block following selective TN block. This is likely due to the proximal spread of local anesthetic that was observed in this investigation and may be occurring in patients with more distal SN bifurcations. Further studies should be aimed at stratifying patients based on bifurcation length and the analgesic benefit of lower volumes of injectate.

We acknowledge several limitations of this study. As mentioned earlier, the tissues of embalmed cadavers differ from in vivo. These differences make ultrasound identification of the nerves more challenging; however, feasibility in embalmed cadavers has been previously established [20]. In the clinical case series, patients with preexisting valgus deformity were excluded because these patients are at higher risk of postoperative peroneal nerve injury. Future investigations could attempt to evaluate SN bifurcation height in this subset of patients to identify candidates who could be safely managed with selective tibial nerve block. However, further studies with larger sample size may be necessary before considering this block in higher risk patients.

An additional limitation was that patients were selected for this study based on availability of the research team. In reviewing Table 1, our study group contained 17 females and 3 males. It is possible that this skewed sample of men and women could have influenced our results. We reviewed twelve months of data from our institution on patients who underwent TKA. There were 561 patients of which 136 were males and 425 were females (76%). Therefore, we felt that the cohort in our case series was consistent with our demographic of patients undergoing TKA. A more even ratio of females to males may have yielded different results; however, this would not have reflected our patient population.

Finally, the anesthesiologist performing the block and collecting the measurements and performing the neurological exam was not blinded and this could have led to a bias. This may have been avoided if a second anesthesiologist was immediately available to measure the local anesthetic and perform the neurological exam. For quality control, we had a second anesthesiologist validate the ultrasound images but this was not available in real-time.

In conclusion, we have demonstrated that selective tibial nerve block at the popliteal crease results in significant proximal spread of local anesthetic toward the SN bifurcation. Furthermore, a shorter bifurcation length is statistically significantly correlated with extension of local anesthetic proximal to the SN bifurcation. This may lead to at least partial blockade of the CPN, which may be undesirable in patients who are at increased risk for peroneal nerve injury after TKA.

## Figures and Tables

**Table 1:** Demographics.

	Mean
Age (years)	67.3 ± (11.1)
Height (cm)	162.2 ± (8.6)
Weight (kg)	77.2 ± (12.8)
BMI (kg/m^2^)	29.5 ± (4)
Leg length (cm)	33.5 ± (4)
Leg circumference (cm)	52.8 ± (7.7)
Distance	
Bifurcation of SN from popliteal crease (cm)	4.8 ± (1.6)
Spread of LA from popliteal crease (cm)^*∗*^	4.7 ± (1.9)
Spread of LA proximal to bifurcation (cm)	−0.0 ± (2.5)

Values expressed as mean and ± standard deviation. ^*∗*^For spread of LA, + values represent spread reaching proximal to the bifurcation and − values represent spread reaching distal to the bifurcation. SN = sciatic nerve; LA = local anesthetic.
